# Supplementary material for: Knowledge, Attitudes, and Perceived Barriers toward Genetic Testing and Pharmacogenomics among Healthcare Workers in the United Arab Emirates: A Cross-Sectional Study
Source: J Pers Med. 2020 Nov 9;10(4):216. doi: 10.3390/jpm10040216 (PMC7711841; doi:10.3390/jpm10040216)
Supplement: Supplementary file 1 [file jpm-10-00216-s001.pdf]

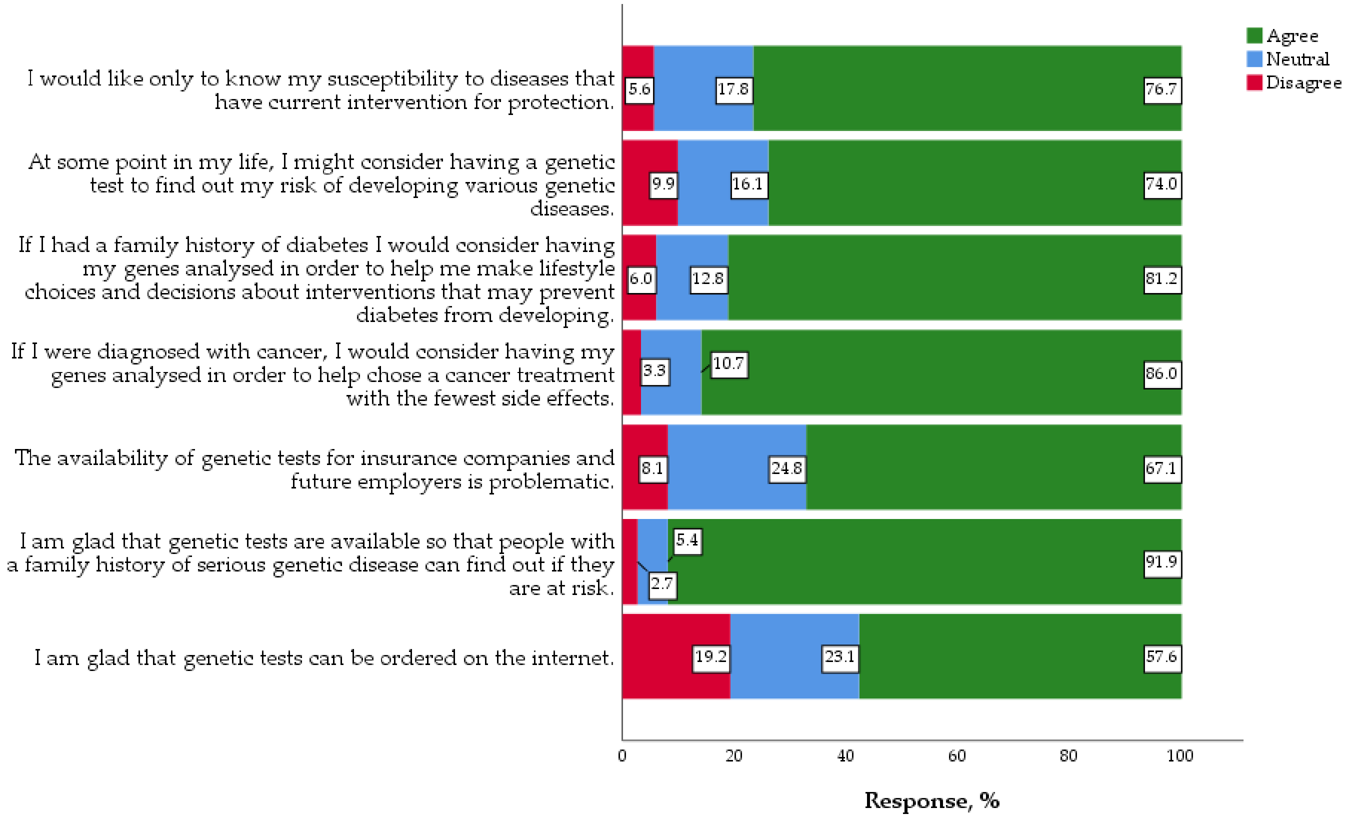

Figure S1 (a): Attitudes on genetic testing (*N* = 388)

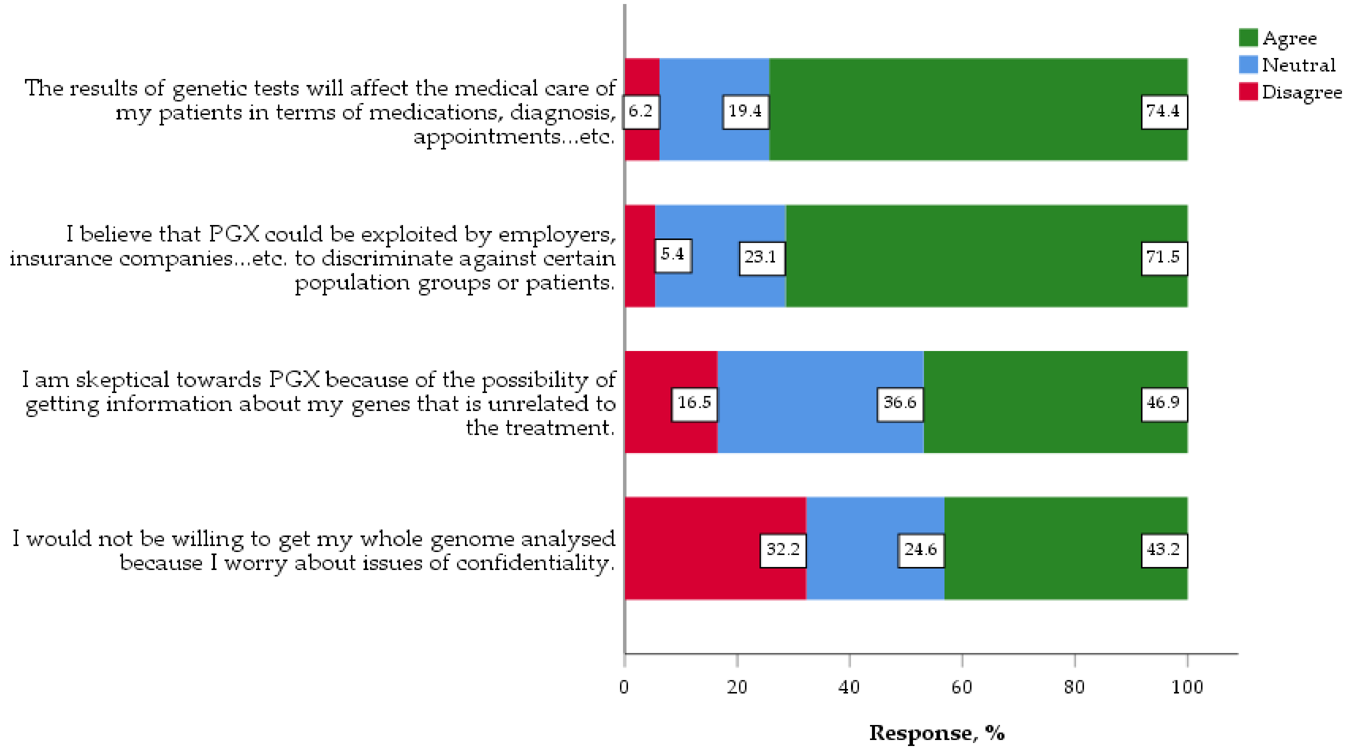

Figure S1 (b): Concerns and ethics on PGX and genetic testing (*N* = 388)
